# Viscosity and Electrical Conductivity of the Liquid Sn-3.8Ag-0.7Cu Alloy with Minor Co Admixtures

**DOI:** 10.1007/s11665-016-2297-8

**Published:** 2016-08-29

**Authors:** A. Yakymovych, V. Sklyarchuk, Yu. Plevachuk, B. Sokoliuk

**Affiliations:** 1 Department of Inorganic Chemistry – Functional Materials, University of Vienna, Währinger Str., 42, 1090 Vienna, Austria; 2 Department of Metal Physics, Ivan Franko National University, Kyrylo and Mephodiy str. 8, Lviv, 79005 Ukraine

**Keywords:** Co admixtures, electrical conductivity, electron, intermetallic, joining, microscopy, viscosity

## Abstract

The viscosity and electrical conductivity as structure-sensitive transport properties of the liquid metals and alloys are important for modeling of the melting and solidification processes. The viscosity and electrical conductivity data provide additional information about the influence of impurities on the structure and physicochemical properties of the liquid metal matrix, which is useful for understanding of structural transformations in the liquid state. In the present work, an impact of minor Co admixtures on the viscosity and electrical conductivity of liquid Sn-3.8Ag-0.7Cu alloy was studied. An increase in viscosity with minor Co admixtures is in a satisfactory agreement with model predicted data obtained from thermodynamic approaches and suggests a significant impact of interatomic interactions. Cobalt admixtures significantly affect the electrical conductivity, which gradually decreases with increasing the amount of Co. Additionally, the sample microstructure has been examined using x-ray diffraction and scanning electron microscopy analyses. The formation of Sn-based Co-Sn intermetallic compounds was detected in the alloys with more than 1 wt.% Co.

## Introduction

The Sn-Ag-Cu alloys (SAC) are widely used as lead-free solders in the electronics industry, and a number of studies intended for enhancement of their characteristics were carried out. Addition of minor amounts of the fourth elements, such as metals in bulk and nanosized forms, ceramic or carbon nanoparticles, is a widespread method to improve mechanical properties of the solder joints (Ref 1-3).

It was recently shown that alloying and impurity metal elements can have several major effects on the reactions between the Sn-based solder and the conductor metal: They can increase or decrease the reaction/growth rate; additives can change the physical properties of the phases formed; finally, they can form additional reaction layers at the interface, or they can displace the binary phases that would normally appear and form other reaction products instead (Ref 4).

A number of investigations were devoted to the effect of different alloying elements as well as impurities on the growth of the intermetallic compound (IMC) layers in Sn-Cu system (Ref 5-10). It was found that addition of appropriate amounts of certain alloying elements to the Sn-based solder can improve the properties of the interfacial compounds, e.g., better drop test reliability, but if excess amounts of the same alloying elements are used, this may cause faster growth of IMCs formed at the interface between solder and substrate, and thus, drastic decrease in reliability can occur (Ref 5).

As an addition to Sn-based solder, cobalt has been attracting a great deal of attention because of its potential benefit. The addition of Co as an alloying element resulted in a better shear ductility of SAC solders, and a reduction in the frequency of the occurrence of brittle failure in ball grid array solder joint improved thermal fatigue and creep resistance and suppression of spalling of interfacial IMC during reflow (see Ref 11 and references therein).

Co belongs to the elements which show marked solubility in IMC layer and has the pronounced effect on IMC formation. It was revealed that small amounts of Co (0.03–0.1 wt.%) change the scallop-type morphology of the Cu_6_Sn_5_ to a more planar one (Ref 12). Co also refines the grain structure of the Cu_6_Sn_5_ layer after reflow and hinders the grain growth of Cu_6_Sn_5_ if subsequent reflows are done. Co dissolves to the Cu sublattice of Cu_6_Sn_5_ [(Cu,Co)_6_Sn_5_] and exhibits negligible solubility to Cu_3_Sn; due to the small grain size and the increased driving force of Sn diffusion through the (Cu,Co)_6_Sn_5_ layer, its growth rate is increased during solid-state annealing. According to Ref 12-15, even very small additions of Co induce beneficial changes in the IMC growth, and thus in the drop test performance, too large amounts of Co tend to decrease the performance of the solder interconnection, owing to an increased growth rate of IMC layers in solid state and because of the pronounced two-phase layer formation during reflow. Anyway, some of these results and their interpretations are ambiguous.

Much less studies are devoted to the influence of minor metal additions on thermophysical properties of liquid Sn-based alloys, in particular, of SAC alloys (Ref 16, 17). Experimental data of structure-sensitive thermophysical properties, such as viscosity and electrical conductivity, are required for mathematical models and simulations describing solidification and soldering processes.

In this work, the influence of minor Co additions on viscosity and electrical conductivity of liquid Sn-3.8Ag-0.7Cu (wt.%) reference alloy (SAC387) has been studied. The experimental viscosity results were compared with data, received from the semi-empirical approaches, namely the Budai–Benko–Kaptay (Ref 18) and Kozlov–Romanov–Petrov (Ref 19) thermodynamic models. The Mott theory (Ref 20) was used for the electrical conductivity analysis. The microstructure of the investigated alloys was analyzed using the x-ray diffraction (XRD) and scanning electron microscopy (SEM).

## Experimental

SAC387 reference alloy and (SAC387)_100−*x*_Co_*x*_ alloys with *x* = 0.5–3 wt.% were synthesized from silver casting grains (99.9% metallic purity, Alfa Aesar, Karlsruhe, Germany), tin ingot (99.998% metallic purity, Alfa Aesar, Karlsruhe, Germany), copper rods (99.9% metallic purity, Alfa Aesar, Karlsruhe, Germany) and cobalt chucks (99.9% metallic purity, Alfa Aesar, Karlsruhe, Germany). Samples were prepared by aging of accurately weighed amounts of the pure components (within ±0.1 mg) for 2 weeks in evacuated and sealed quartz ampoules at 1173 K.

The viscosity measurements of liquid SAC387 reference alloy and (SAC387)_100−*x*_Co_*x*_ alloys were taken using a high-temperature oscillating-cup viscometer (Ref 21). According to this method, a cylindrical graphite crucible containing the sample with a mass of about 15 g is placed in a stainless steel container attached to the torsion wire inside the high-temperature furnace. The experiments were performed in an argon atmosphere. The viscometer was three times evacuated with a pump before the measurements. The temperature was determined by WRe-5/20 thermocouples. Each sample was heated up to 1073 K and kept at this temperature at least 1 h for homogenization. The viscosity was measured during cooling from the highest temperature with a constant cooling rate of about 4 K/min.

The viscosity was calculated from the logarithmic decrement and the period of oscillations using the modified Roscoe equation (Ref 22). After each measurement, the weight of the sample was checked. In all cases, the loss of material by vaporization was lower than 0.2% of the ingot. The resultant error of the viscosity measurements did not exceed 3%.

The electrical conductivity measurements were taken by the 4-point method in an argon atmosphere. Graphite electrodes for current and potential measurements were placed in the wall of the vertical cylindrical boron nitride ceramic measuring cell along its vertical axis. The potential electrodes were provided with thermocouples for temperature measurements. Single thermoelectrodes of these thermocouples were used for electrical conductivity determination. The melt temperature was determined by WRe-5/20 thermocouples located in close contact with the liquid. For further details of this method and its experimental realization, we refer to Ref 23. Each sample was inserted into the cell directly inside a high-pressure vessel. Thus, the actual sample composition was accurate within a tolerance of 0.02 wt.%. The resultant error of the electrical conductivity measurements is about 2%.

After viscosity measurements, the alloy samples were analyzed by scanning electron microscopy (SEM) and x-ray diffraction. Metallographic investigations were performed using the electron microscope Zeiss Supra 55 VP. The excitation energy of the electron beam was 20 kV; the surfaces of the samples were visualized by the detection of backscattered electrons. The chemical analyses of the sample phases were performed using the energy-dispersive x-ray (EDX) technique with four characteristic spectral lines of Co, Cu (K-line) and Ag, Sn (L-line). Pure Co was also used for energy calibration of EDX detector signal. Standard deviations for the chemical compositions obtained from EDX were about ±1 at.%.

The powder XRD measurements were taken on a Bruker D8 diffractometer at ambient temperature using Ni-filtered CuK_α_ radiation (accelerating voltage 40 kV, electron current 40 mA). The diffractometer operates in the *θ/2θ* mode. The powder was fixed with petroleum jelly on a single-crystal silicon sample carrier which was rotated during the measurement. The detection unit was a Lynxeye strip detector. Indexing of the phases was supported by the Inorganic Crystal Structural Database (ICSD). Rietveld refinement of the XRD patterns was done with the Topas3^®^ software provided by Bruker AXS.

## Theoretical Predictions

A number of thermodynamic approaches are widespread used for viscosity calculations of liquid metal alloys. Two of the most common thermodynamic models for viscosity predictions were chosen in the presented work, namely the Kozlov–Romanov–Petrov model and the Budai–Benkö–Kaptay model.

### Kozlov–Romanov–Petrov Model

Kozlov et al. proposed a simple equation for the calculation of the viscosity of liquid alloys including the integral enthalpy of mixing of the alloy, Δ_*mix*_*H*, and viscosities of the components (Ref 19):

1 $$ \ln \eta = \sum\limits_{i = 1}^{n} {x_{i} } \ln \eta_{i} - \frac{{\Delta _{\text{mix}} H}}{3RT}, $$

where *x*_*i*_ and *η*_*i*_ are the concentration and viscosity of the component *i*, respectively; *T* is the absolute temperature; and *R* is the ideal gas constant.

### Budai–Benkö–Kaptay Model

The equation for the viscosity of liquid multi-component alloys according to the Budai–Benkö–Kaptay model is expressed as (Ref 24):

2 $$ \eta = A\frac{{\left( {\sum\limits_{i} {x_{i} } M_{i} } \right)^{1/2} }}{{V^{2/3} }}T^{1/2} \exp \left[ {\frac{B}{T}\left( {\sum\limits_{i} {x_{i} T_{m,i}^{ * } } - \frac{{\Delta_{mix} H}}{qR}} \right)} \right], $$

where *M*_*i*_ is the molar mass of the component *i*; *q* is a semi-empirical parameter equal to *q* ≅ 25.4 ± 2 (Ref 25); *V* is the molar volume of the alloy; Δ*H* is the enthalpy of mixing; *A* and *B* are fitting parameters equal to (1.80 ± 0.39) × 10^−8^ (J/(K mol^1/3^))^1/2^ and (2.34 ± 0.20), respectively; and *T*_*m*,i_^*^ is the effective melting temperature of the component *i*:

3 $$ T_{m,i}^{ * } = \frac{T}{B}\ln \left( {\frac{{\eta_{i} V_{i}^{2/3} }}{{AM_{i}^{1/2} T^{1/2} }}} \right), $$

where *V*_*i*_ is the molar volume of the component *i*.

In our calculations, the excess volume of the alloys investigated is assumed to be equal to zero. The enthalpy of mixing was taken from Ref 26; the density, viscosity and atomic volume of the components were taken from Ref 27-29.

## Results

**Fig. 1:**
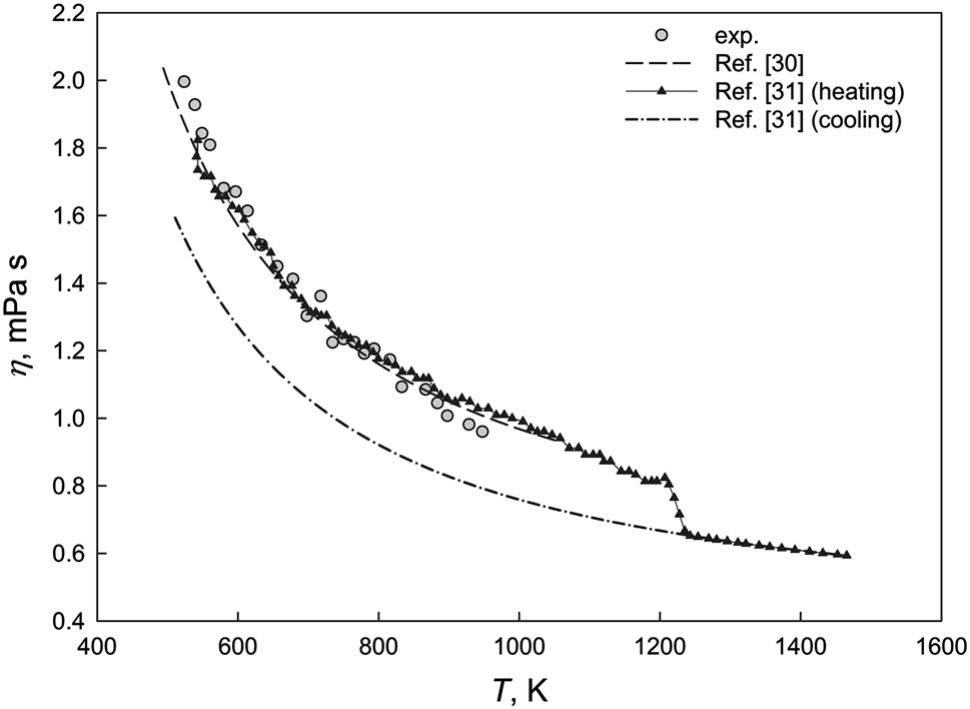
Temperature dependence of the viscosity of liquid SAC387 reference alloy

Figure 1 shows a temperature dependence of the experimental viscosity for the liquid SAC387 (Ag_3.8_Cu_0.7_Sn_95.5_) alloy together with the literature data (Ref 30, 31). The viscosity values from Ref 31 were digitized.

Our experimental data are in agreement with Ref 30 and the heating curve *η*(*T*) below 1073 K given by Rozhitsina et al. (Ref 31). In contrast to viscosity behavior of Sn, and eutectic Ag-Sn, Cu-Sn and Ag-Cu-Sn alloys described in Ref 31, we did not reveal any hysteresis and points of branching of the heating and cooling viscosity curves.

**Fig. 2:**
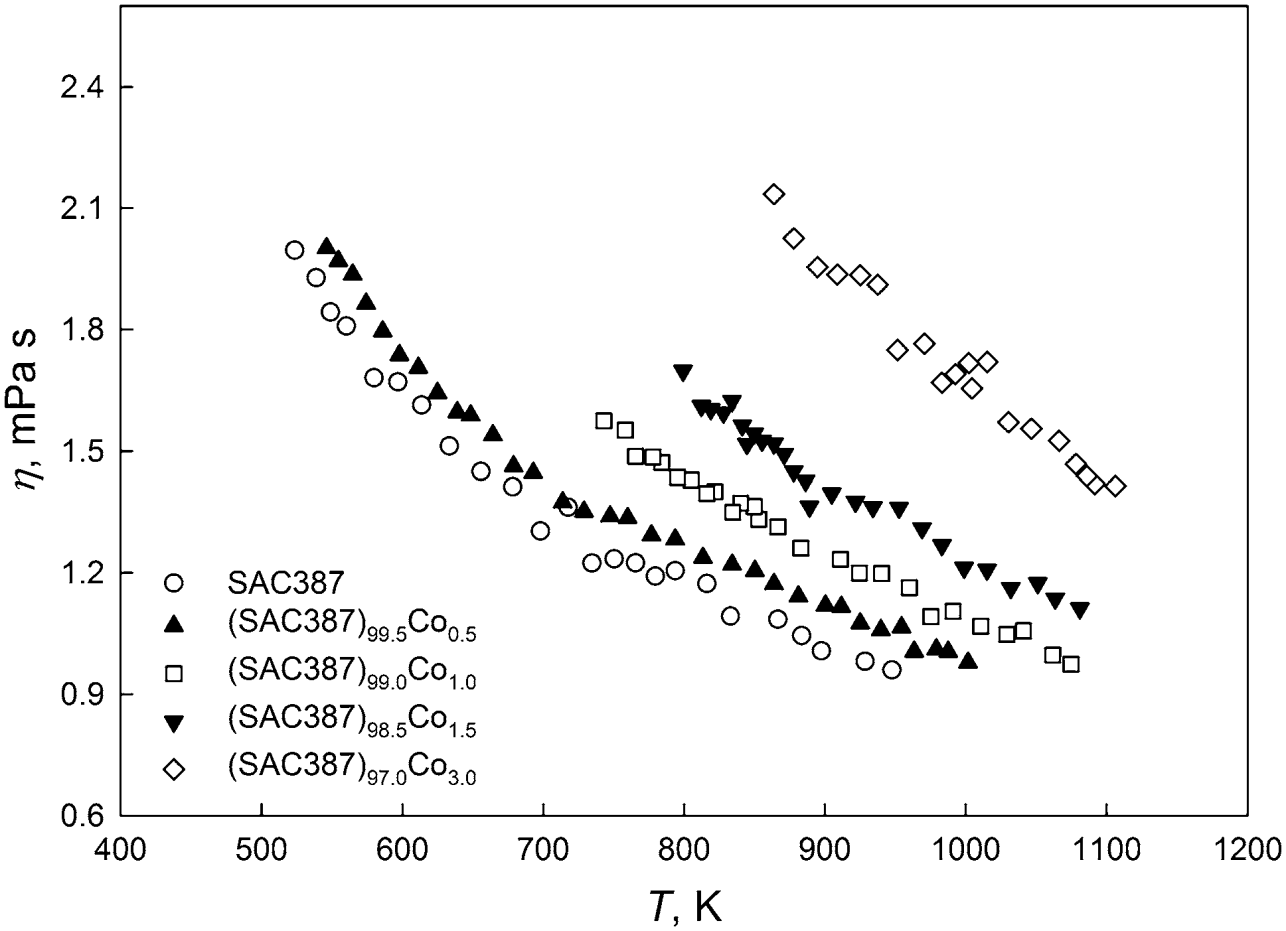
Temperature dependence of the viscosity of liquid (SAC387)_100−*x*_Co_*x*_ alloys

The viscosity of liquid (SAC387)_100−*x*_Co_*x*_ alloys increases upon cooling according to the Arrhenius-type equation (Fig. 2):

4 $$ \eta = \eta_{0} \exp \left( {\frac{{E_{\eta } }}{RT}} \right), $$

where *η*_0_ is a constant; *E*_*η*_ is the activation energy of the viscous flow; *T* is the absolute temperature; and *R* is the ideal gas constant. The values of *E*_*η*_ and *η*_0_, determined by the least square regression fits of the experimental data according to Eq 4, are presented in Table 1.

**Table 1:** Fitting numerical parameters of the Arrhenius-type Eq 4

Alloy composition, wt.%	*η*_0_, mPa s	*E*_*η*_, 10^3^ J/mol
SAC387	0.448	6.28
(SAC387)_99.5_Co_0.5_	0.444	6.85
(SAC387)_99.0_Co_1.0_	0.357	9.27
(SAC387)_98.5_Co_1.5_	0.356	10.31
(SAC387)_97.0_Co_3.0_	0.342	13.14

**Fig. 3:**
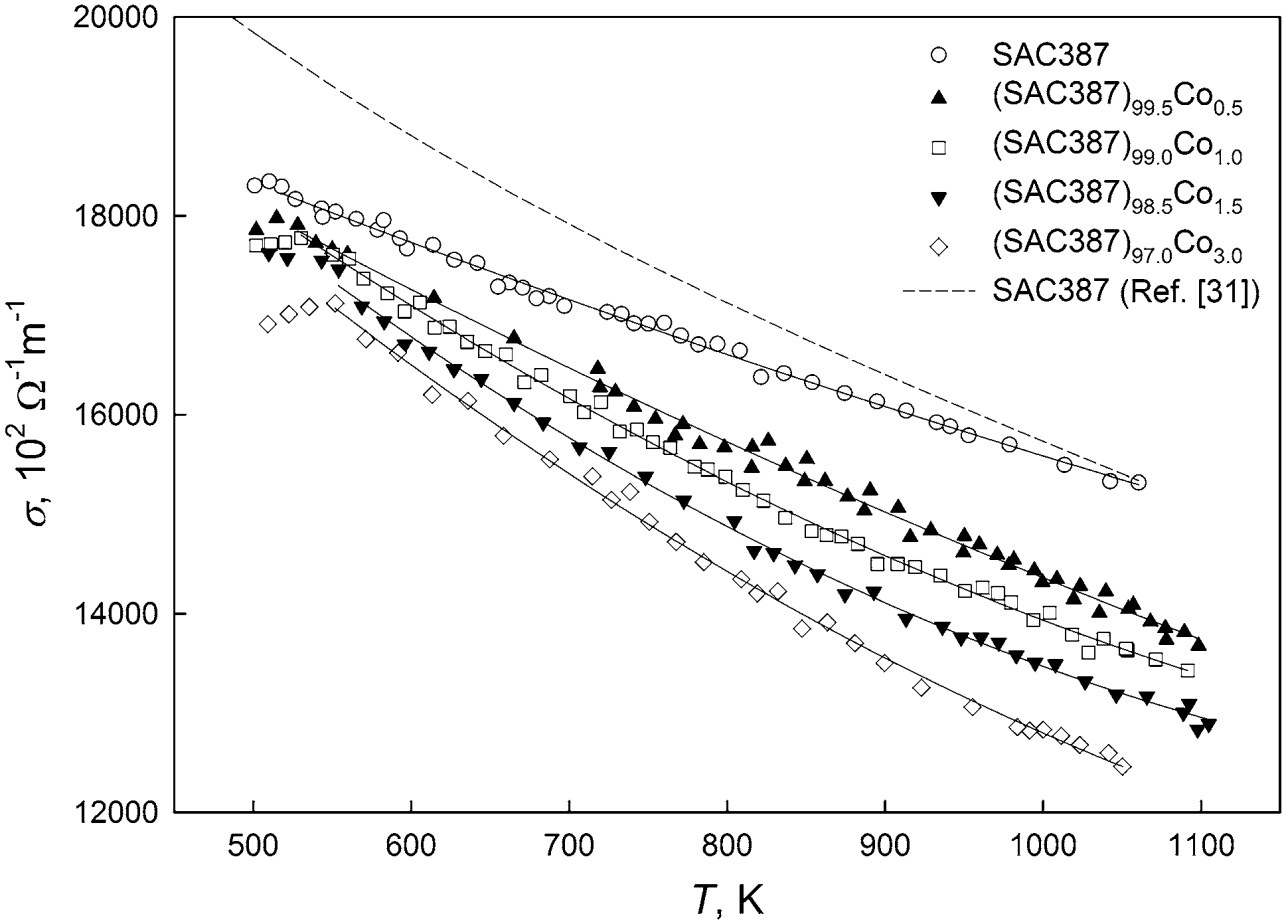
Temperature dependence of the electrical conductivity of liquid (SAC387)_100−*x*_Co_*x*_ alloys

Temperature dependence of the electrical conductivity *σ*(*T*) of liquid alloys SAC387, (SAC387)_99.5_Co_0.5_, (SAC387)_99.0_Co_1.0_, (SAC387)_98.5_Co_1.5_ and (SAC387)_97.0_Co_3.0_ was measured during heating and cooling in the temperature range between 500 and 1100 K. A gradual conductivity decrease upon heating was observed for all the alloy compositions (Fig. 3), and for each concentration, the experimental data have been fitted by second-order polynomials, namely:

5 $$ \sigma = A + B_{1} T + B_{2} T^{2} . $$

The coefficients of the polynomials are listed in Table 2.

**Table 2:** Coefficients of the polynomials adjusted on the experimental conductivity for different alloy compositions (from 500 to 1100 K)

Alloy composition, wt.%	*A*, 10^2^/Ω m	*B*_1_, 10^2^/Ω m K	*B*_2_, 10^2^/Ω m K^2^
SAC387	21,749	−7.4882	0.0013
(SAC387)_99.5_Co_0.5_	22,855	−10.5778	0.0021
(SAC387)_99.0_Co_1.0_	24,710	−15.5590	0.0048
(SAC387)_98.5_Co_1.5_	25,532	−18.3549	0.0063
(SAC387)_97.0_Co_3.0_	25,448	−18.3011	0.0057

A slope of the *σ*(*T*) curve of SAC387 differs from that determined in Ref 30. A deviation between the absolute conductivity values from this study and data of Ref 30, which is noticeable just after melting, vanishes at higher temperatures.

As illustrated in Fig. 3, the addition of Co significantly affects the electrical conductivity, which gradually decreases with increasing the amount of Co addition. It can be seen (Fig. 3) that (SAC387)_97.0_Co_3.0_ alloy with the highest cobalt content exhibits the lowest conductivity.

## Discussion

**Fig. 4:**
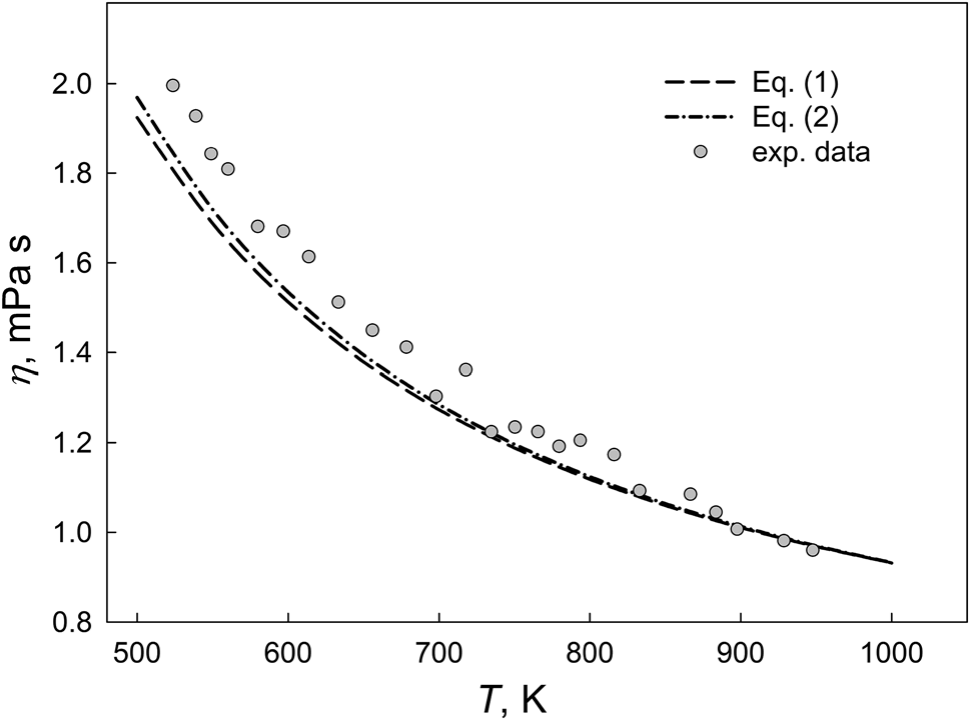
Comparison of the calculated and experimental viscosity data for liquid SAC387 alloy

The temperature dependencies of the viscosity for liquid SAC387 alloy calculated according to the Kozlov–Romanov–Petrov and Budai–Benkö–Kaptay approaches are in a good agreement with experimental data (Fig. 4). The maximal difference between the experimental and calculated data from Eq 1 did not exceed 6%.

In order to examine a possibility to apply for quaternary alloys the chosen thermodynamic predictions and the calculated and experimental viscosity were compared at 1073 K. As given in Table 3, the experimental values exceed the data calculated from thermodynamic approaches, and this difference increases with an increase in the cobalt content. Based on the presented data, a satisfactory agreement between calculated viscosity values and experimental data is obtained.

**Table 3:** Predicted viscosity values using Eq 1 and 2 for (SAC387)_100−*x*_Co_*x*_ alloys by the comparison with experimental data (*η*_exp_) at 1073 K

Alloy composition, wt.%	*η*_exp_, mPa s	*η*_(1)_, mPa s	*η*_(2)_, mPa s
SAC387	0.90	0.91	0.90
(SAC387)_99.5_Co_0.5_	0.96	0.93	0.92
(SAC387)_99.0_Co_1.0_	1.01	0.95	0.94
(SAC387)_98.5_Co_1.5_	1.13	0.98	0.95
(SAC387)_97.0_Co_3.0_	1.49	1.05	1.00

The analysis of the electrical conductivity is based on the assumption that an addition of admixtures decreases the electrical conductivity, and this conductivity decrement can be described by the following equation:

6 $$ \Delta \sigma_{i}^{ - 1} = N_{i} \frac{{mv_{F} }}{{e^{2} }}\varSigma_{i} , $$

where Σ_*i*_ is the scattering cross section of the conducting electrons at the admixture particles; *N*_*i*_ is the atomic fraction of these impurities; *m* is the electron mass; and *v*_*F*_ is the electron velocity at the Fermi level (Ref 20). It was revealed for different systems that in a simplest case, when the impurity scattering does not depend on other scattering mechanisms, ∆*σ*_*i*_ is temperature independent (Ref 32, 33).

We believe that the scattering process in the (SAC387)_100−*x*_Co_*x*_ alloys is the same. At the same time, some anomalies in the conductivity behavior were revealed in the temperature range close to solidification. As shown in Fig. 3, the cooling of the samples is accompanied by a gradual nonlinear conductivity increase.

Approaching the solidification temperature, a change in the *σ*(*T*) curves has been observed. It is suggested that the cobalt admixtures dissolved in the basic matrix at high temperatures. Thus, the eutectic composition has been shifted, and during the solidification, a hypereutectic alloy was formed. The latter is characterized by two temperatures, *T*_*L*_ and *T*_*S*_, which correspond to the beginning and the end of solidification, respectively.

In order to examine the microstructure of investigated alloys after viscosity and electrical conductivity measurements, a few selected samples were analyzed by SEM-EDX and x-ray diffraction. The results of the phase analysis along with BSE images of three selected alloys are given in Table 4. According to the obtained results, no residual pure Co was found in the samples. The XRD phase analysis fully confirmed no pure Co in samples after viscosity measurements that had been found by SEM-EDX. Meanwhile, the Co atoms substitute the atoms of Cu in Cu_6_Sn_5_ compound. Similar substitution was observed by Gao et al. (Ref 34) studying the morphology and grain growth pattern of intermetallic compounds formed at the interface between Sn-3.5Ag-0.1Co (wt.%) solder and the Cu substrate. In Ref 35, it was found that the composition of Cu_6_Sn_5_ intermetallic in the (Ag_3.5_Co_0.1_Sn_96.4_)/Cu diffusion couple is (Cu_90_Co_10_)_6_Sn_5_ and (Cu_99_Co_1_)_6_Sn_5_, which is in agreement with our results presented in Table 3. Furthermore, Cu substitutes Co atoms in CoSn_3_ IMCs, what is in an agreement with the literature data in Ref 36. Moreover, in the investigated alloys with more than 1 wt.% Co, the CoSn_2_ and CoSn phases were found.

**Table 4:** SEM–EDX results of (SAC387)_100−*x*_Co_*x*_ samples after viscosity measurements

Sample	Phase 1		Phase 2			Phase 3			Phase 4				Phase 5				Co-Sn phases			SEM image
		Sn, at.%		Ag, at.%	Sn, at.%		Cu, at.%	Sn, at.%		Co, at.%	Cu, at.%	Sn, at.%		Co, at.%	Cu, at.%	Sn, at.%		Co, at.%	Sn, at.%	
SAC387	βSn	100	Ag_3_Sn	60	40	Cu_6_Sn_5_	52	48												
BA1	βSn	100	Ag_3_Sn	75	25	Cu_6_Sn_5_	52	48	(Co,Cu)_6_Sn_5_	1	49	50								
BA2	βSn	100	Ag_3_Sn	75	25	Cu_6_Sn_5_	52	48	(Co,Cu)_6_Sn_5_	2–6	50–43	48–50	(Co,Cu)Sn_3_	20	4	75	CoSn_2_	32	68	
BA3	βSn	100	Ag_3_Sn	75	25	Cu_6_Sn_5_	50	50	(Co,Cu)_6_Sn_5_	3	48	49	(Co,Cu)Sn_3_	20	4	76	CoSn_2_ CoSn	36 49	64 51	
BA4	βSn	100	Ag_3_Sn	75	25	Cu_6_Sn_5_	49	51	(Co,Cu)_6_Sn_5_	3	48	49	(Co,Cu)Sn_3_	23	1	76	CoSn_3_ CoSn_2_ CoSn	25 33 49	75 67 51	

BA1—bulk (SAC387)_99.5_Co_0.5_ alloy

BA2—bulk (SAC387)_99.0_Co_1.0_ alloy

BA3—bulk (SAC387)_98.5_Co_1.5_ alloy

BA4—bulk (SAC387)_97.0_Co_3.0_ alloy

## Conclusions

The minor cobalt admixtures to the SAC387 alloy gradually increase the viscosity and decrease the electrical conductivity. These effects might be caused by changes in the interatomic interactions, which lead to formation of the new phases during solidification. Such suggestions are confirmed by the microstructure analysis of the samples performed after viscosity measurements. It was shown that Co atoms replace the atoms of Cu in Cu_6_Sn_5_ compounds when small additions of Co (up to 1 wt.%) are added to the SAC387 reference alloy. Further increase in the Co content results in formation of the Sn-based Co-Sn compounds in the alloy. Formation of the new Co-Sn phases in the SAC387 alloy with Co additions should reinforce the microstructure in the solid state, and enhanced mechanical properties of SAC387 similar to Sn-based Sn-Cu and Sn-Ag-Cu solders reinforced with In, Ni or Zn, respectively (Ref 37, 38).
